# A Stochastic Step Model of Replicative Senescence Explains ROS Production Rate in Ageing Cell Populations

**DOI:** 10.1371/journal.pone.0032117

**Published:** 2012-02-16

**Authors:** Conor Lawless, Diana Jurk, Colin S. Gillespie, Daryl Shanley, Gabriele Saretzki, Thomas von Zglinicki, João F. Passos

**Affiliations:** 1 Institute for Cell and Molecular Biosciences, Newcastle University, Newcastle-upon-Tyne, United Kingdom; 2 Centre for Integrated Systems Biology of Ageing and Nutrition, Newcastle University, Newcastle-upon-Tyne, United Kingdom; 3 Institute for Ageing and Health, Newcastle University, Newcastle-upon-Tyne, United Kingdom; 4 School of Mathematics & Statistics, Newcastle University, Newcastle-upon-Tyne, United Kingdom; Keio University, Japan

## Abstract

Increases in cellular Reactive Oxygen Species (ROS) concentration with age have been observed repeatedly in mammalian tissues. Concomitant increases in the proportion of replicatively senescent cells in ageing mammalian tissues have also been observed. Populations of mitotic human fibroblasts cultured *in vitro*, undergoing transition from proliferation competence to replicative senescence are useful models of ageing human tissues. Similar exponential increases in ROS with age have been observed in this model system. Tracking individual cells in dividing populations is difficult, and so the vast majority of observations have been cross-sectional, at the population level, rather than longitudinal observations of individual cells.

One possible explanation for these observations is an exponential increase in ROS in individual fibroblasts with time (e.g. resulting from a vicious cycle between cellular ROS and damage). However, we demonstrate an alternative, simple hypothesis, equally consistent with these observations which does not depend on any gradual increase in ROS concentration: the Stochastic Step Model of Replicative Senescence (SSMRS). We also demonstrate that, consistent with the SSMRS, neither proliferation-competent human fibroblasts of any age, nor populations of hTERT overexpressing human fibroblasts passaged beyond the Hayflick limit, display high ROS concentrations. We conclude that longitudinal studies of single cells and their lineages are now required for testing hypotheses about roles and mechanisms of ROS increase during replicative senescence.

## Introduction

Since the proposal of the Free Radical Theory of Ageing [1], reports linking Reactive Oxygen Species (ROS) generation, mitochondrial function and the ageing process have been accumulating [2]. However, despite this wealth of mostly correlative evidence, we are still far from understanding the role of ROS in ageing. Recent experimental observations have reopened the question of the role of ROS in the ageing process; observations of ROS concentrations and fraction of senescent cells after genetic interventions affecting both mitochondrial function and oxidative stress in animal models have led to conflicting conclusions [3], [4], [5], [6].

The role of ROS in replicative senescence, the permanent arrest of the cell cycle in normally proliferating cells such as fibroblasts, is not well understood.

Early evidence suggested that cells which were continuously passaged *in vitro* until senescence underwent time-dependent accumulation of damage. Senescent cell populations were found to have increased production of ROS [7], [8] and to accumulate oxidation products such as protein carbonyls, protein oxidative modifications [9], lipofuscin [10], [11] and DNA damage [8]. Mitochondria, the main sources of ROS, have been implicated in the process, since senescent cells were found to have impaired metabolism [8], [12] and damage to mitochondria [8].

More importantly, interventions which decreased ROS in cells were found to impact on replicative senescence. Exposure to low ambient oxygen concentrations [13], [14], free radical scavengers [15], [16], overexpression of antioxidant enzymes [17] and mild, chronic mitochondrial uncoupling [8] have been shown to decelerate telomere shortening and to extend replicative lifespan of cells in culture. These data are all consistent with the hypothesis that one of the major contributing factors to replicative senescence is time-dependent accumulation of oxidative damage.

However, evidence has emerged suggesting a novel role for ROS in replicative senescence. It has been reported that the activation of key players in the senescence pathway contributes to ROS generation, without cells undergoing time-dependent damage accumulation. Signalling via Ras [18], p53 [19], p21 [20], [21] and p16 [22] have been shown to induce ROS generation, contributing to the initiation of the senescent phenotype. These observations could be explained by ROS acting as signalling molecules and therefore as outcomes of a tightly regulated process signalling for replicative senescence [23], [24].

However, one question remains: how can we reconcile data suggesting a gradual ROS increase preceding induction of senescence with the observation that ROS increases in a rapid, step-wise manner as a consequence of signalling events, similar to apoptosis?

Cross-sectional observations of ROS and ROS-derived products in populations of cells at different Population Doublings (PDs) are consistent with a hypothesis of a gradual, cumulative increase in ROS generation. However, cell populations are highly heterogeneous [25] and cells bearing senescent markers can be found even in populations of young cells [8], [26]. Here we examine an alternative biological hypothesis to gradual cellular-level changes in phenotype: that the change in ROS observed at the population-level is a mere consequence of a time-dependent increase in the fraction of senescent cells in the population, with individual cells undergoing discrete, asynchronous, stochastic increases in ROS levels. In order to have a mechanistic understanding of the role of ROS in replicative senescence, we must be able to distinguish between these two very different hypotheses.

To test our alternative hypothesis we developed a Stochastic Step Model of Replicative Senescence, describing discrete, stochastic changes in cellular ROS levels upon transition from proliferation to replicative senescence. We present a dataset describing increasing rates of Mitochondrial ROS production with increasing PD in populations of human MRC5 fibroblasts, repeatedly passaged until they reach replicative senescence. By comparing experimental observations with model predictions we demonstrate that the gradual, continuous ROS dynamics observed in these populations could also be explained by stochastic, discrete transitions between proliferative and senescent states at the cellular level without any recourse to gradual accumulation of damage in individual cells. In other words, the increase of ROS observed during long-term cultivation of primary cells could be a consequence of the senescent phenotype rather than a gradual accumulation of damage as earlier proposed. We demonstrate that, in mixed populations of senescent and proliferating cells, those cells which are not expressing senescence markers (Ki67+ve with less than 5 DNA damage foci [26]) have significantly lower rates of ROS production than those which do not. We also present a second dataset demonstrating that cellular ROS production rates remain constant in proliferation-competent cells (MRC5 fibroblasts overexpressing *hTERT*) passaged 90 PDs beyond the Hayflick limit, which is not consistent with a hypothesis of gradual accumulation of damage in cells, but is consistent with our alternative hypothesis and our model.

## Materials and Methods

### A step model of changing phenotypes in an ageing cell population

**Figure 1:**
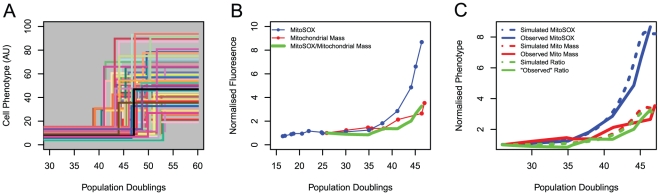
A simple stochastic step model explains Reactive Oxygen Species production in cell populations undergoing senescence and in immortal cells. A) Hypothesis: Schematic representation of a step increase in phenotype for 100 cells. Each coloured trace represents one cell as it undergoes transition from proliferation-competence to senescence. Phenotype for each cell in the proliferating state is drawn from a relatively narrow distribution; whereas the phenotype of senescent cells is drawn from a broader distribution, with a higher expected value. All phenotype transitions are strictly non-decreasing. With increasing PD a higher proportion of cells in the population become senescent. B) Normalised, mean MitoSOX fluorescence measured by flow cytometry varies with PD in MRC5 fibroblasts cultivated until senescence (blue points). Normalised mean mitochondrial mass in a distinct population of the same cell type varies with PD (red points). Linear interpolation approximations to these curves were constructed (red and blue lines) and MitoSOX/mitochondrial mass estimated (green line); C) Kinetics of interpolated (solid lines) normalised MitoSOX fluorescence (blue), normalised mitochondrial mass fluorescence (red) and MitoSOX/mitochondrial mass ratio (green) are compared with mean population estimates from a stochastic step model (dashed lines). Stochastic step model parameters are estimated from the distributions for a wholly proliferative population (PD_27_) and for a wholly senescent population (PD_47_).

We assume that cellular phenotypes change in a discrete fashion during the transition from proliferation-competence to senescence (see Figure 1A). The size, morphology, division rate or oxidative state of cells could change in this fashion for example. Figure 1A illustrates a proliferating population of cells with a relatively narrow distribution of phenotypes undergoing stochastic step transitions to a much broader distribution of phenotypes associated with replicative senescence. The population doubling number at which this transition takes place is also sampled from a distribution, representing variability in the actual age of individual cells as well as variability in the age at which they undergo transition. These three distributions capture biological heterogeneity in states and in timings which are not actively coordinated.

Here we consider a general phenotype of value *ϕ*. We describe the expected *ϕ* for a mixed population of senescent and proliferation-competent cells: *ϕ_Mix_*. We assume that there are two underlying *ϕ* distributions corresponding to sub-populations of proliferation-competent and replicatively senescent cells, with an expected value for proliferation-competent cells: *ϕ_P_*, and a distinct expected value for replicatively senescent cells: *ϕ_S_*. In this analysis we estimate these two parameters from experimental observations of populations of cells which we assume to be 100% proliferating and 100% senescent respectively.

We can then describe *ϕ_Mix_* as a function of the fraction of proliferating cells (*p*) and the fraction of senescent cells (*s*):
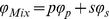
(1)We assume that there are only senescent and proliferating cells in the population, therefore:
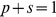
(2)Further, we assume that the senescent phenotype value is greater than that of the proliferating phenotype:
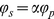
(3)

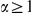
Substituting Eqs. 3 and 2 into Eq. 1 gives:
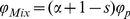
(4)The fraction of senescent cells found in a population can be estimated as a function of population doublings: *s(PD)*, from an observed growth curve for that population as described previously [26], or by observing the change in fraction of cells in a population expressing biomarkers for senescence with PD. The former method has two advantages over the latter: it provides an estimate of senescent fraction consistent with the most fundamental definition of replicative senesence (an irreversible loss of cell division potential) as well as providing an estimate of uncertainty in senescent cell fraction for all PDs. We therefore use the growth curve method for estimating senescent cell fraction dynamics in this analysis. The analysis presented previously was carried out on the same cell line we investigate here [26]. We constructed a linear interpolation function *s(PD)* from hundreds of parametric estimates generated previously between PD_25_ and PD_47_ and constructed an estimate for *ϕ_Mix_* as a function of population doubling number:
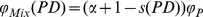
(5)An equivalent expression can be derived for the variance of *ϕ* subject to assumptions about the covariance between phenotype distributions for proliferating and senescent cells.

We selected MitoSOX/mitochondrial mass ratio (*MSMM*) rate as our phenotype *ϕ*, expressing it as a function of population doubling number as in Eq. 5. To compare model predictions with observed data, we estimated *ϕ_P_* as the mean normalised ROS production rate observed in a population of cells which are known to be >95% proliferating (e.g. a population with low doubling number (<30), Figures 1B, 1C). α is estimated as *ϕ_S_*/*ϕ_P_*, where *ϕ_S_* is similarly estimated from a population of cells known to be ∼100% senescent (e.g. a population with doubling number close to the Hayflick limit which has stopped dividing). Model predictions for intermediate, mixed populations were plotted and compared with experimental observations (Fig. 1C) using the R statistical software package [27].

### Phenotype distribution analysis

We fitted Gamma distributions to microscopically observed MitoSOX levels per cell from a proliferating cell population, and from a wholly senescent population (Figure S1A). A mixed population of 50% proliferating and 50% senescent cells consistent with the stochastic step model was simulated. The simulated mixture was tested for bimodality with 500 samples (representing a practical, experimentally observable population size) using Hartigan's dip test [28] as implemented in the *diptest* package in R.

We performed a similar analysis on samples from WI-38 cell volume distributions observed by Mitsui & Schneider [29]. We digitised the observed cell volume frequency distributions for proliferating cells (PD_19_) and fully senescent cells (PD_45_) using GraphExtract2.5 for Windows. We converted these to probability density distributions and sampled from these repeatedly to simulate mixed populations of senescent and proliferating cells consistent with the step model and tested simulated mixed populations for bimodality as above (Figure S1B).

We also investigated whether predicted population distribution changes could be adequately predicted by the SSMRS. To do this, we considered MitoSOX fluorescence alone as our phenotype *ϕ*. Measurements of thousands of cells were made by fluorescence-activated cell sorting of populations of our MRC5 fibroblasts, capturing distributions of MitoSOX fluorescence per cell at PD_38_, PD_41_, PD_44_, PD_45_ and PD_46_. We assumed that all cells at PD_38_ were proliferation competent and used this distribution as *ϕ_P_*. We can see from Figure S2F that about 90% of cells were proliferation competent at that PD and therefore this assumption is reasonable. We also assumed that the observed population at PD_46_ was completely senescent, and therefore used this distribution as *ϕ_S_*. Again, we can see from Figure S2F that this is reasonable. We also assumed that the uncertainty in senescent fraction as a function of PD (*s(PD)*, Fig. S2 F) which was previously estimated for these cells [26] comes from stochasticity in the timing of cellular transition from proliferation competence to replicative senescence. For intermediate PDs (PD_41_, PD_44_ and PD_45_) we simulated 10,000 cells, assigning a probability that cells were proliferating or senescent, based on the uncertainty in *s(PD)*, Fig. S2 E. We then randomly classified each simulated cell as proliferating or senescent, weighted by those probabilities. For each cell classified as proliferating, we sampled (with replacing) a MitoSOX fluorescence observation from *ϕ_P_*. Similarly, for each cell classified as senescent, we sampled a MitoSOX fluorescence observation from *ϕ_S_*. We then plotted the simulated mixed populations alongside observed intermediate distributions and observed good agreement (simulated fluorescence was greater than observed fluorescence for only 45%, 51% and 53% of the 10,000 simulated samples at intermediate PDs: PD_41_, PD_44_ and PD_45_ respectively).

### Cell culture

Human embryonic lung MRC5 fibroblasts were obtained from ECACC (Salisbury, UK) and cultured until reaching senescence. Cells were considered senescent when positive for Sen-β-Gal and negative for proliferating markers (BrdU and Ki67).

MRC5 cells were transfected retro-virally at PD_30_ with the human catalytic subunit (*hTERT*) of the enzyme telomerase as described in [30]. These transfected cells were cytogenetically tested at PD_100_. No cytogenetic abnormalities were observed.

Mouse ear fibroblasts (MEFs) were obtained from C57Bl6 wild-type mice at 3 months of age.

All cells were grown in DMEM supplemented with 10% foetal calf serum, 2 mM glutamine and 1% pen/strept under controlled conditions (air plus 5% CO_2_) in a 3-gas incubator. Cells were always passaged before confluency.

### Ethics statement

All work complied with the guiding principles for the care and use of laboratory animals. The project was approved by the Faculty of Medical Sciences Ethical Review Committee, Newcastle University. Project licence number 60/3864.

### MitoSOX staining and Immunofluorescence

Flow cytometric analysis of MitoSOX and NAO fluorescence was performed as described in [8].

Cells grown on coverslips were stained with 5 µM MitoSOX (Invitrogen) for 10 min at 37°C. Cells were washed with PBS and fixed with 2% paraformaldehyde for 5 min.

Immunofluorescence was then conducted as described in [8]. Anti-γH2A.X mouse monoclonal (Upstate), Anti-Ki-67 Rabbit polyclonal (ab15580 Abcam) were used.

All fluorescence microscopy was performed using a Leica DM5500B fluorescence microscope, using a 40×1.3 NA oil objective. Fluorescent images were always taken under identical excitation and emission condition. MitoSOX intensity was quantified using ImageJ (http://rsb.info.nih.gov/ij/). For each experiment a minimum of 50 cells were analysed.

## Results

### A simple stochastic step-model explains the rate of ROS production in a population of cells undergoing replicative senescence

We cultured MRC5 fibroblasts from PD_18_ until replicative senescence (PD_47_) and measured average population MitoSOX fluorescence by flow cytometry. We observed an exponential increase in population MitoSOX fluorescence with PD (Figure 1B). A proportion of this increase is due to increases in mass of mitochondria per cell during the transition from proliferation to senescence [31], and so we also measured mitochondrial mass by NAO fluorescence in a separate sub-population of the same cell line. To estimate MitoSOX/mitochondrial mass ratio (*MSMM*) we approximated each of the fluorescence time courses from distinct, replicate cell populations by linearly interpolating between observations (Figure 1B & C), normalising each by their value at the earliest common PD (PD_27_), giving estimates of fluorescence kinetics across the full range of observed PDs. We constructed MSMM from separately normalised MitoSOX and mitochondrial mass observations. We modelled *MSMM* dynamics with the stochastic step model described above, setting *ϕ_P_ = MSMM* observed at PD_27_ and *ϕ_S_* = *MSMM* observed at PD_47_. We repeated this analysis for normalised MitoSOX and mitochondrial mass separately. Modelled population mean MitoSOX fluorescence, mitochondrial mass and *MSMM* increased in an approximately exponential manner with PD (Figure 1B). Calibrating model parameters with observations from young (proliferative) cells and old (wholly senescent) cell populations, and using an estimate of the rate of increase of senescent cell fraction with PD [26], model predictions for all three of these phenotypes were validated by comparison with experimental observations at intermediate PDs (Fig. 1C). Experimental observations were adequately described by the stochastic step model: Pearson's correlation coefficient between observed and simulated mean *MSMM* were >0.93 for all three comparisons.

MitoSOX staining distributions measured by flow cytometry as cells reach senescence were unimodal at any given analysed time point (Fig. S2 A–D). Intuitively the absence of a bimodal distribution in a mixed population of both young cells (with low MitoSOX staining) and senescent cells (with high MitoSOX staining) could seem inconsistent with a step-wise increase in ROS and might be suggest that cells with intermediary levels of ROS exist at any given time-point before 100% of the population undergoes senescence.

To test if this observation was consistent with our step-model we simulated MitoSOX distributions of a population containing a mixture of proliferating (50%) and senescent (50%) cells by mixing experimental observations from cell populations containing predominantly proliferating cells (PD_20_) and from populations of senescent cells (PD_47_) and analysing the resultant mixture distributions.

The simulation revealed that such populations still showed a unimodal MitoSOX distribution (Figure S1A). This is likely to be a consequence of senescent populations being more heterogeneous than proliferating ones (standard deviation more than 8 times greater), greater even than the increase expected from the increase in distribution mean (less than 7 times greater).

An independent analysis using data on cell volume distributions from another study [29] confirms that simulated distributions of mixed populations of senescent and proliferating cells are entirely consistent with an unimodal distribution (Figure S1B).

We also found that changes in the shape of population phenotype distribution could be adequately predicted by the SSMRS (Figure S2).

### Ki67 negative cells containing more than 5 γH2A.X foci have significantly higher MitoSOX levels than Ki67 positive cells

Previously we have shown that the absence of activity of the proliferation marker Ki67 combined with high density of DNA damage foci (>5 foci/nucleus) was a quantitative marker of senescence in both human and mouse fibroblasts [26]. We have now developed an experimental method which allows simultaneous assessment of MitoSOX fluorescence together with immunofluorescence against Ki67 and γH2A.X.

**Figure 2:**
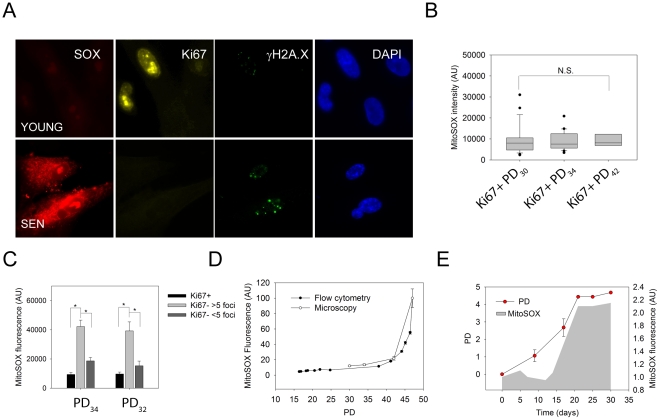
Ki67 negative cells containing more than 5 γH2A.X foci have significantly higher MitoSOX levels than Ki67 positive cells. A) Representative fluorescent images of YOUNG (PD_30_) and senescent (SEN, PD_47_) cells stained with MitoSOX followed by immunostaining against Ki67 and γH2A.X. MitoSOX staining (red); Ki67 (yellow); γH2A.X (green); DAPI (blue). B) Boxplots showing MitoSOX intensity in Ki67+ fibroblasts at 3 different PDs (∼50 cells per condition were quantified) C) MitoSOX intensity in Ki67+, Ki67−<5 γH2A.X foci and Ki67−>5 γH2A.X foci MRC5 fibroblasts (∼50 cells per condition were quantified). Asterisk indicates significance when comparing Ki67−>5 foci with the two other groups by 2-Way ANOVA; D) Comparison between MitoSOX fluorescence kinetics obtained by flow cytometry and microscopy in human fibroblasts; E) MitoSOX data (grey area) and growth curve (red circles) of mouse ear fibroblasts grown under 20% oxygen (data are from 3 independent mice).

Firstly, we performed MitoSOX staining on MRC5 fibroblasts at various PDs (e.g. Fig. 2A) and quantified fluorescence intensity per cell (Figs. 2B & C). We found similar results to the ones found by flow cytometry, confirming that we can reliably quantify MitoSOX fluorescence using microscope images (Figure 2D).

Secondly, we combined MitoSOX staining and immunofluorescence against Ki67 and γH2A.X (Figure 2A–C). We observed that MitoSOX intensity remained constant with PD in Ki67 positive cells (Figure 2C). In the low percentage of Ki67− cells with more than 5 γH2A.X foci which we found in pre-senescent cultures, MitoSOX fluorescence levels were substantially higher than in Ki67 positive cells or in Ki67− cells with <5 γH2A.X foci (Figure 2C).

The same exponential increase in MitoSOX fluorescence, consistent with the step model, was confirmed in fibroblasts isolated from C57Bl6 wild-type mice ears. During the proliferative stage, no differences in MitoSOX staining were observed. Once the fibroblasts reached senescence a two-fold increase in MitoSOX fluorescence was observed (Figure 2E).

### hTERT overexpressing MRC5 cells do not undergo an exponential increase in ROS production rate despite being cultured throughout 140 population doublings

**Figure 3:**
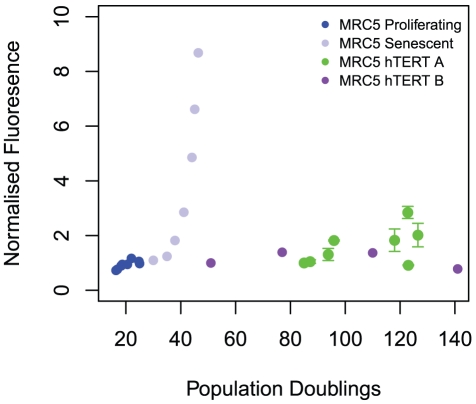
Mitochondrial ROS does not increase significantly with population doublings following *hTERT* overexpression. Mean MitoSOX fluorescence measured by flow cytometry at increasing PDs in a purely proliferative population of MRC5 fibroblasts (blue points), in the same population of cells undergoing transition to senescence (light blue points), and in two independent experiments using MRC5 fibroblasts immortalised by transfection with hTERT (green points & purple points). Fluorescence in MRC5 fibroblasts is normalised according to the value at PD_27_, as in Fig. 1B. All other datasets are normalised according to the fluorescence levels at the earliest observed PD.

We ectopically overexpressed *hTERT* in human MRC5 fibroblasts at PD_30_ [30]. The resulting cells displayed high levels of telomerase activity, stabilized telomere length and greatly extended replicative lifespan [30]. We measured MitoSOX fluorescence systematically and found no significant changes occurring over a wide range of PDs (Figure 3). A low, constant level of MitoSOX, approximately equal to that observed in non-transfected MRC5 fibroblasts, is not consistent with damage accumulation with time leading to increased ROS but is entirely consistent with the stochastic step-model which we present here.

## Discussion

During *in vitro* studies, cross-sectional samples of passaged cell populations are taken after various population doublings. Cell age is typically classified on a population-level by PD, indicating the mean number of divisions undergone since an initial progenitor cell. However, it has been extensively described that cell populations are heterogeneous in terms of their division potential, even in clonal populations derived from single progenitors [25]. The heterogeneity in division potential, which is inherently stochastic, is evident from the presence of cells bearing senescent markers even in young populations [26].

As cells reach senescence, a progressive increase in ROS generation can be observed [7], [8], [12]. Most literature has suggested oxygen toxicity as a major contributor to ROS generation during *in vitro* senescence. The inability of cells to deal with atmospheric oxygen concentration has been proposed as a major contributor to the time-dependent accumulation of several types of damage, such as lipofuscin, mitochondrial dysfunction, protein oxidation and DNA damage [11], [32], [33].

Recently, it has been demonstrated that ROS were necessary for the long-term maintenance of DDR and for the stability of growth arrest which characterises the senescent phenotype [21]. It is therefore possible that prolonged *in vitro* cultivation does not lead to time-dependent ROS generation in proliferation-competent cells and that the majority of ROS generated is a consequence of processes which occur secondarily to cells becoming irreversibly arrested.

Our observations are consistent with such a concept. Firstly, proliferating cells (whether immortalised, and grown for extended passages beyond the Hayflick limit, or simply positive for proliferating marker Ki67) appear to be unchanged in terms of time-dependent ROS accumulation. Secondly, a model simulating a step-like ROS increase in senescent cells is able to explain population kinetics of ROS generation during serial cultivation of populations of human fibroblasts.

Other factors linked to senescence will also contribute to this ROS increase. The fact that senescent cells are larger and contain more mitochondria [31] will contribute to the increase in MitoSOX fluorescence observed in senescent cells. Nonetheless, we have confirmed that the increase in MitoSOX fluorescence is still significantly higher in senescence when we normalise either for mitochondrial mass or cell size.

Although our model can explain the major changes occurring during cultivation, we cannot invalidate damage accumulation as a contributing factor to the induction of senescence, since it has been well demonstrated that interventions reducing oxidative damage can extend replicative lifespan and reduce fractions of cells bearing senescent markers [34]. One possibility is that damage does accumulate but at lower levels than we can detect using the available assays. In this case, our data suggest that mitochondrial ROS could be a useful marker of senescence.

The model we present here is distinct from the hypothesis that ROS increase is a consequence of time-dependent damage accumulation in proliferating cultured fibroblasts. While both hypotheses are consistent with cross-sectional, population average experimental observations from fibroblasts undergoing the transition to senescence, new experimental observations presented here, particularly ROS immutability in proliferation-competent cells in old cell populations and in hTERT-overexpressing cells dividing well past the Hayflick limit, strongly support our stochastic step model.

Further experimental dissection of these alternate hypotheses might greatly improve our understanding of the ageing process since cells expressing various senescence markers, including DNA damage foci, do accumulate in human [35], primate [36], [37] and mouse [38], [39] tissues with advancing age. Moreover, in mice tissues, age-dependent increase in fraction of senescent cells has been shown to be temporally and spatially associated with markers of oxidative stress [38], [40]. In the livers of ageing mice, around 20% of hepatocytes are positive for γH2A.X DNA damage foci which have been shown to correlate with the Sen-β-Gal senescence biomarker. Even higher frequencies of γH2A.X positive cells have been found in small intestinal crypts, lungs and spleens of old mice [38].

The association of senescence with a sudden, large increase in ROS production might be responsible for the negative impact of senescent cells on their tissue environment and contribute to organismal ageing. However, one needs to be cautious when extrapolating results from *in vitro* models to the *in vivo* context, as further experiments need to be conducted to ascertain the role of ROS produced by senescent cells *in vivo*.

Nonetheless, supporting this hypothesis is the finding that mice with critically short telomeres, the late generation TERC−/−, exhibit increased fraction of senescent cells [41], as well as increased ROS [21] in tissues. Moreover, deletion of CDKN1A, one of the downstream effectors of cellular senescence, is able to reduce ROS production and extend lifespan in late generation TERC−/− mice [21], [42].

To fully discriminate between a hypothesis of gradual accumulation of ROS during senescence and our alternate hypothesis of discrete stochastic changes in heterogeneous populations, and to develop our understanding of the causes and consequences of replicative senescence, longitudinal studies of single cells and their lineages are now required.

## Supporting Information

Figure S1 **The stochastic step model of replicative senescence predicts unimodal phenotype distributions for mixed populations of cells undergoing transition to senescence.** A) MitoSOX fluorescence per cell quantified from microscopy images for a proliferating population (PD_20_, red histogram) and a wholly senescent population (PD_47_, blue histogram) of MRC5 fibroblasts. MitoSOX levels for both populations were modelled as following gamma distributions (red and blue curves respectively). A mixed population of 500 cells (50% senescent) was simulated by sampling from the two distribution models and the kernel density estimate for the mixed population is shown here (black dashed line). 500 is a reasonable emulation of a cell population which can practically be observed. B) Frequency distributions from [29] describing the volume of WI-38 cells (human foetal lung fibroblasts) at PD_19_ and PD_45_ were digitised and replotted here after conversion to probability density distributions (red and blue solid lines respectively). Assuming the step model describes the change in cell volume in these cells, simulating mixed populations of proliferating and senescent cells by weighted random sampling from distributions from PD_19_ and PD_45_ does not produce bimodal cell volume distributions (as an example, a 50∶50 mixture of senescent and proliferating cells is simulated, dashed black curve).
(EPS)

Figure S2 **The stochastic step model of replicative senesence predicts the shape of MitoSOX distributions for mixed populations of cells undergoing transition to senescence.** A) MitoSOX fluorescence density distribution obtained by flow cytometry of MRC5 fibroblasts at PD_37.9_. Approximately 90% of cells at this PD were proliferation competent (see panel F); B–D) For PD_41.1_ (B) PD_44.1_ (C) and PD_45.1_ (D) we simulated mixtures of 10,000 cells, assigning a probability that cells were proliferating or senescent by analysis of cell growth curve (see panel F) and randomly sampling from distributions at PD_37.9_ and PD_46.4_ depending on whether cells were classified as proliferating or senesecent. Density distributions show that the simulated mixed populations (red line) and experimental data obtained by flow cytometry (black line) are in good agreement; E) MitoSOX fluorescence distributions obtained by flow cytometry of MRC5 fibroblasts at PD_46.4_. The majority of these cells at this PD were senescent. F) Senescent cell fraction dynamics estimated from analysis of growth curves. Solid black line is mean cell fraction estimate and dashed lines represent 95% confidence range. Vertical blue lines represent PDs for model calibration distributions (A,E). Vertical red lines represent PDs for intermediate model validation distributions (B–D).
(PDF)
