# Supplementary figures and images for: A Stochastic Step Model of Replicative Senescence Explains ROS Production Rate in Ageing Cell Populations
Source: PLoS One. 2012 Feb 16;7(2):e32117. doi: 10.1371/journal.pone.0032117 (PMC3281103; doi:10.1371/journal.pone.0032117)

**A**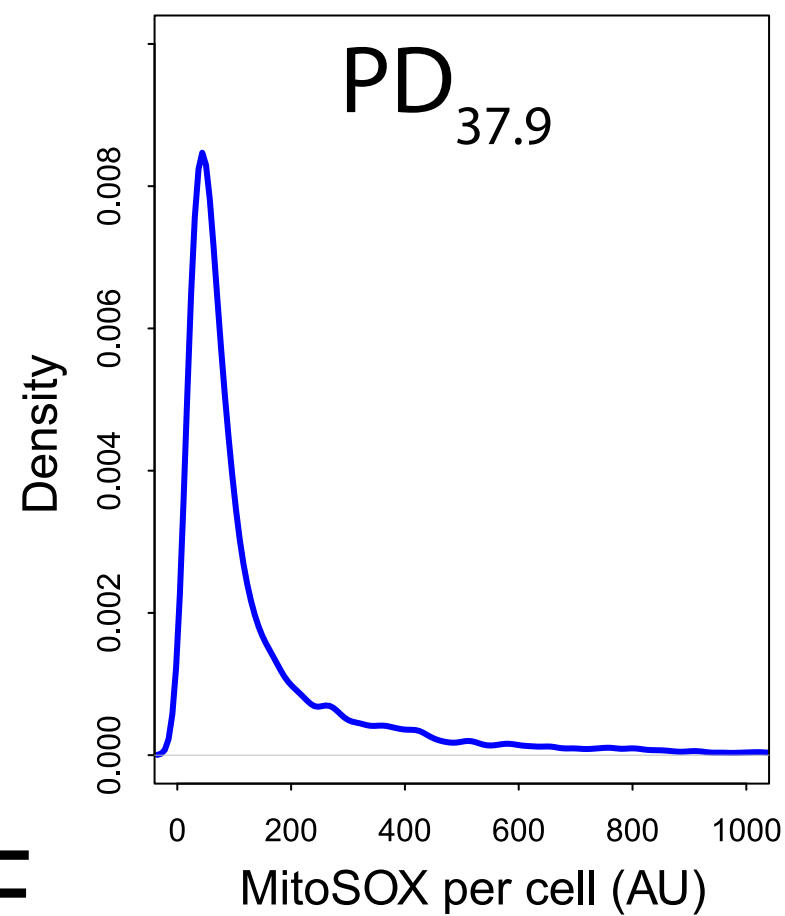**B**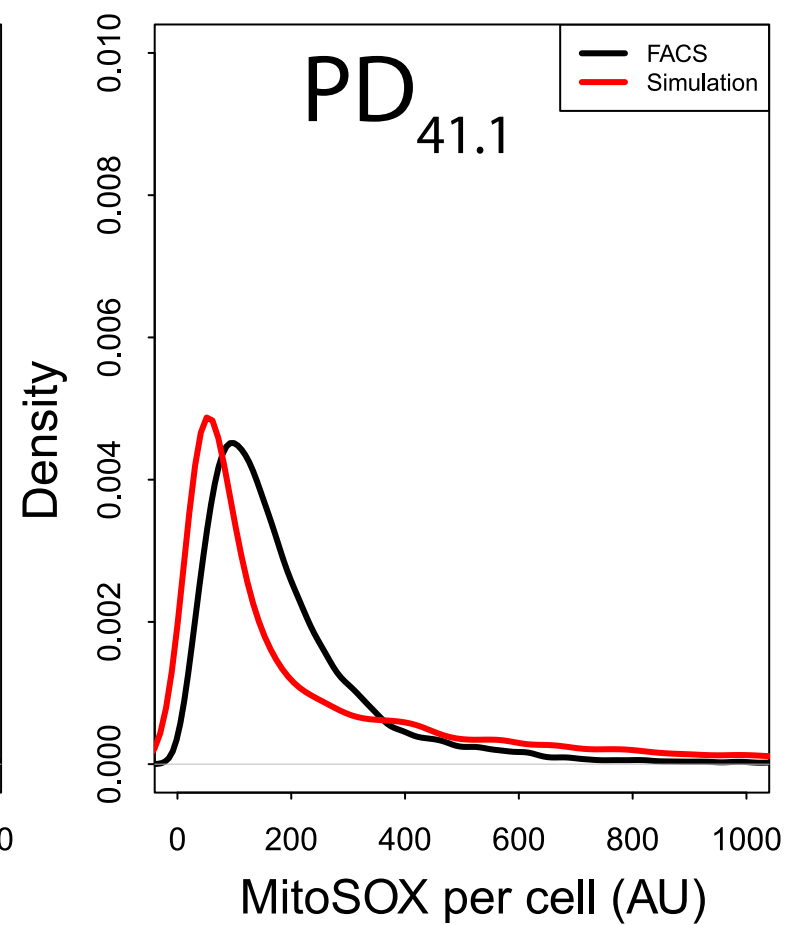**C**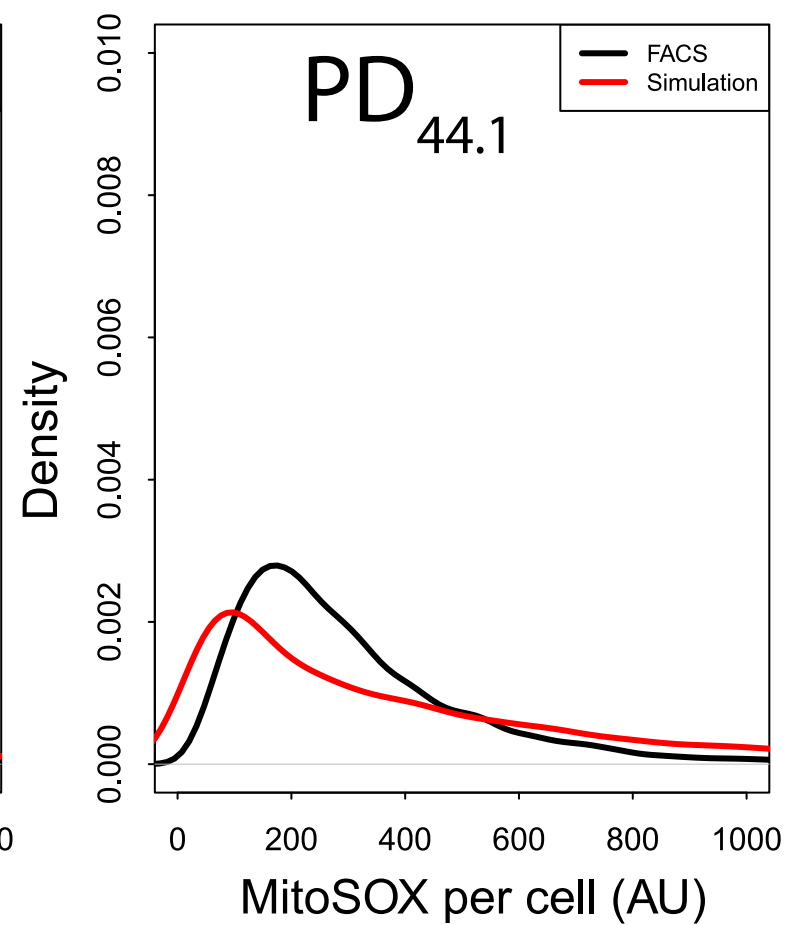**D**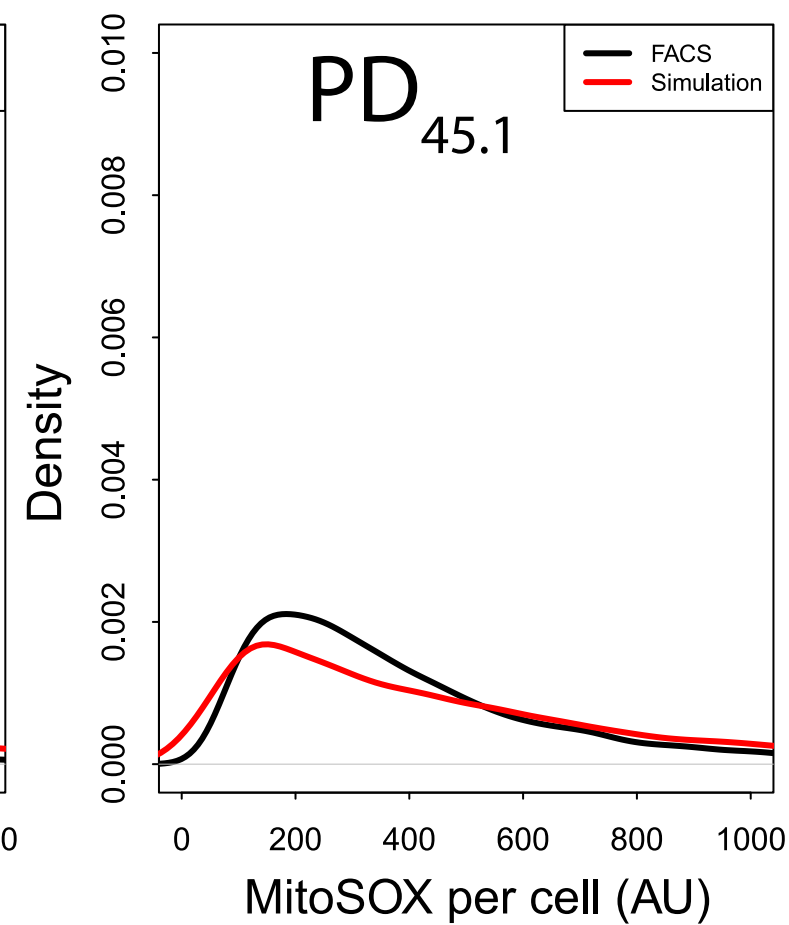**E**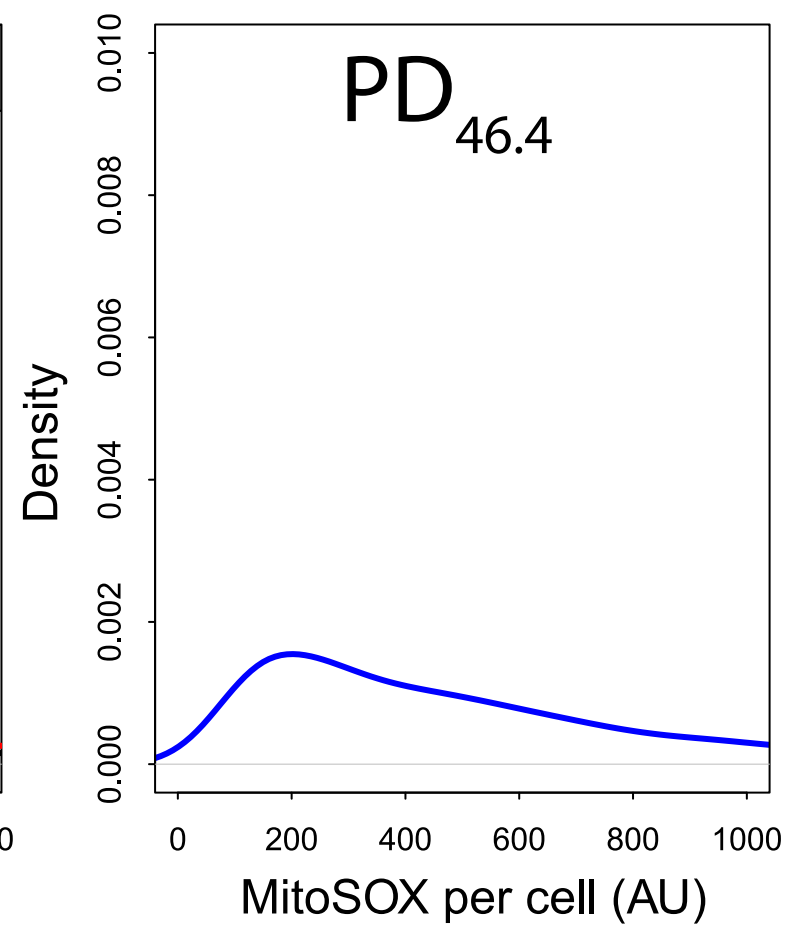**F**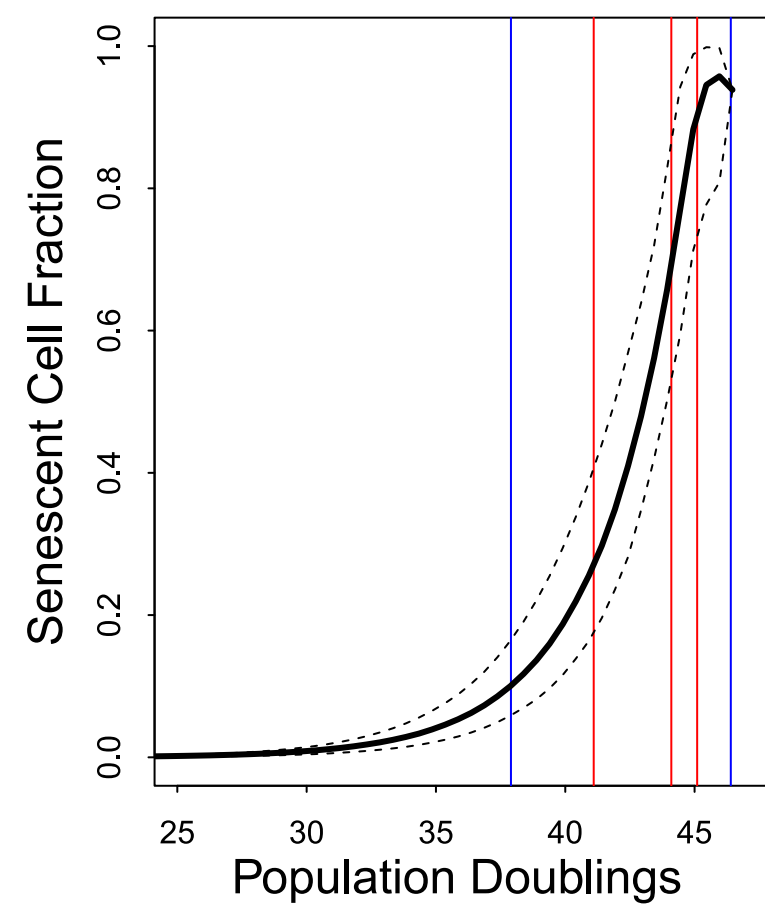

Supplement: Figure S2 — The stochastic step model of replicative senesence predicts the shape of MitoSOX distributions for mixed populations of cells undergoing transition to senescence. A) MitoSOX fluorescence density distribution obtained by flow cytometry of MRC5 fibroblasts at PD37.9. Approximately 90% of cells at this PD were proliferation competent (see panel F); B–D) For PD41.1 (B) PD44.1 (C) and PD45.1 (D) we simulated mixtures of 10,000 cells, assigning a probability that cells were proliferating or senescent by analysis of cell growth curve (see panel F) and randomly sampling from distributions at PD37.9 and PD46.4 depending on whether cells were classified as proliferating or senesecent. Density distributions show that the simulated mixed populations (red line) and experimental data obtained by flow cytometry (black line) are in good agreement; E) MitoSOX fluorescence distributions obtained by flow cytometry of MRC5 fibroblasts at PD46.4. The majority of these cells at this PD were senescent. F) Senescent cell fraction dynamics estimated from analysis of growth curves. Solid black line is mean cell fraction estimate and dashed lines represent 95% confidence range. Vertical blue lines represent PDs for model calibration distributions (A,E). Vertical red lines represent PDs for intermediate model validation distributions (B–D). (PDF) [file pone.0032117.s002.pdf]
